# An Account of the Disease Which Was Epidemic in Some Parts of New-York and New-England, in the Winter of 1812–13

**Published:** 1815-02

**Authors:** 


For the Medical and Physical Journal.

### *An Account of the Disease which was Epidemic in some Parts of New-York and New-England, in the Winter of 1812–13.*

"AMERICA," say the learned editors of the [late] London Medical Review, "seems to be the country of epidemics, as much as it is of swamps, woods, and savannahs." England, we might say in return, seems to be the country of scrophula and consumption, as much as it is of fogs and vapours. Such observations are at least injudicious, not to say incorrect. America has never suffered in proportion half so much from the severity of epidemic diseases as England. The yellow fever has been confined to two or three large cities, and the spotted fever to districts of country not very thickly populated; and the mortality from these disorders, compared to the whole population of the country, has been of trifling importance. We have never yet seen thousands falling by the plague, or the sweating sickness. The diseases of America are fewer in number, and more acute, than those of Europe. They are principally fevers and inflammations. We see but little of gout and dropsy, and scrophula, and the long train of chronic complaints, which vex the inhabitants, and enrich the physicians, of the older countries. The number of deaths, compared to the whole population, is probably smaller than that of any European country. We find, for example, on comparing the proportion of deaths to the whole population, in the *town* of Boston, with the proportion of deaths to the whole population of France, according to the statistical accounts of Peuchet, that while in the latter there dies annually one person in thirty, in the former there is about one only in forty-eight. This exhibits a great difference in favour of the salubrity of our climate, especially when we observe, that the comparison is between a populous closely settled town and a whole territory, comprehending both town and country, a country acknowledged to be the most healthy in Europe. In truth, it is difficult to explain the rapid increase of population in the northern section of the United States, except it be admitted to be an uncommonly healthy climate. In the New-England States, we have perhaps as few foreigners as any nation in Europe. There are no emigrations to our already populous country, but, on the contrary, there is a constant emigration to the interior of New York, Pennsylvania, and the North West territory, besides the current which commerce pours out over the whole face of the earth.

Notwithstanding

Notwithstanding this loss, it appears, that, from the year 1800 to 1810, the population of Massachusetts and Maine, which compose one state, was increased from 574,564 to 700,745, while the states into which our emigrations took place, have increased in a greater proportion.—What the writer means by swamps and savannahs, we do not exactly know, unless he believes the *prairies* of the Mississippi to extend to the Atlantic; or supposes the great lakes to be stagnant pools. But for the information of all Europeans, who have not studied the geography of America, we beg leave to make known, that, except the sea coast of the southern states, which consists of alluvial soil, the country is rather hilly; and that the New England states, to which the remarks alluded to most specially apply, form one of the most uneven rocky mountainous countries on the face of the globe. The illiberality and want of information displayed by the author quoted above, have elicited these remarks; but we wish to be understood as meaning no disrespect to English writers generally. It is from them we receive a considerable part of what we know, and we, of course, ought to pay them the tribute of our gratitude and veneration. There are, on both sides of the water, men of narrow and illiberal minds, who are provoked by prejudice and jealousy to slander those they ought to respect and love, as descendants from the same ancestors.

We have been imperceptibly drawn away from the subject of this paper, the epidemic that has appeared in New-York and New-England during the last winter. It is proposed to give a concise account of the symptoms of this disorder, its treatment, and the morbid appearances after death.

This complaint made its first appearance in the autumn of 1812, among the soldiers at Greenbush, and since that time its ravages have been remarkable in the army of the United States, in various places; at Plattsburgh, Burlington, Sacket's Harbour, Boston, Charlestown, &c. Its severity in the army is to be attributed to the sudden change of the mode of living of the newly enlisted soldiers, to intemperance, and to exposure to the weather. During the winter, it appeared among the citizens in Vermont, and the upper part of New-York, and at the close of winter in some of the interior towns of Massachusetts, and sporadically in various parts of the United States. Its fatality among the soldiers, and, in a few places, among the citizens, was considerable; but, so far as can be ascertained, it has not, in most places, increased the number of deaths beyond the usual proportion. The victims of this disease in Boston may have been between twenty and thirty in number, of which about half were soldiers.

soldiers. It is impossible to estimate the numbers affected with various forms of the complaint in the same place. They could not have been less than four or five hundred.

In the most characteristic cases, the disease commences with an acute pain in the side or breast, stricture across the thorax and difficulty of breathing, short distressing cough, chills, great prostration of strength, pulse a little accelerated, rather full, sometimes hard, but easily arrested by pressure. The tongue soon becomes yellow, then brown and dark-coloured, the countenance peculiarly livid, and the patient slightly delirious. Afterwards the cough increases, expectoration sometimes occurs, and the patient gets relieved on the third, fourth, fifth, or sixth day in some instances, while in others the disease runs on irregularly for some days longer. The heat of the body is greater than usual, but never approaches that of common inflammations. The skin is often moist from the beginning, both in mild and very bad cases. In many instances, the patient is first attacked with pain in the head, back, and limbs, severe chills, and very depressed pulse. In the worst cases, the pain in the side or head is dreadful, there is no cough, the stricture in the chest is agonizing, the patient becomes delirious, and dies in two or three days. Sometimes the complaint resembles a common pleurisy, except that the pulse is not so hard, the cough nor the heat so great, nor the progress so regular. A young officer, living in barracks, where a number of soldiers had died very suddenly of the disorder, having been exposed to cold and severe exertion, was attacked with pain in the left side and sickness at the stomach, at midnight. In the morning, on attempting to get out of bed, he fell on the floor. Being removed to a more comfortable situation, he was bled six ounces, and thus relieved from the pain in the side. The blood had a remarkable inflammatory buff, yet the pulse was not above 80, full, and very compressible. After this, an emetic was administered, which produced an evacuation of a small quantity of very dark-coloured mucus. His restlessness increased during the day, and at night the pain returned, for which a blister was applied to the side. The next morning, his tongue, at first pale, was very brown, and afterward became dry and dark-coloured. He had a slight cough, a stricture over the thorax, pulse, as at first, full yet compressible, pain in the side severe and distressing, excessive restlessness, and a degree of delirium. Six ounces of blood were taken, which relieved the pain in the side. He had small doses of calomel and opium every two or three hours; and, no evacuation from the bowels having occurred since the invasion of disease, a cathartic was ordered at noon, noon. This did not operate till the following morning. On the third day, the pain was not heard of, the restlessness abated; and, on the fourth, all the symptoms were mitigated, without any remarkable evacuation. Calomel and opium were continued a day or two longer. The patient convalesced slowly at first, but got out of the house in a fortnight from his attack, and has since joined the army.

When the heart was the principal seat of disease, which happened sometimes, an intense pain struck the region of that organ, together with great palpitations, irregularity, depression, and annihilation of the pulse, syncope, and cold sweats; but in some cases, the pulse was very rapid, and not depressed. When the heart and lungs were affected together, there was a mixture of symptoms, though the symptoms of disease in one organ commonly predominated. Very frequently the abdominal region seemed to be the seat of disease. Then there was a pain and stricture across that part, attended sometimes with diarrhœa, and oftener with costiveness. Sometimes the head was most violently affected. The patient, seized with an intense pain in that part, soon became delirious, and died. This rarely occurred in our part of the country; but we have frequently witnessed cases of acute pain in one eye. These different appearances were accompanied with a peculiarity of pulse, such as before indicated, an irregular increase of heat, and a moist skin. They usually yielded to medical treatment with sufficient facility, except those remarkable cases of the head and heart. All these are to be considered as variations from the common appearance of the complaint.

Some physicians have been so fortunate as to be able to distinguish various stages of the disorder; and to suppose they could determine its critical and non-critical days. We must confess that we have distinguished nothing but want of uniformity and regularity, and an utter defiance of the laws which govern other diseases. Some cases were of the duration of a few hours, others of weeks; some were very slight, others very severe; some were attended with considerable heat and elevation of the pulse, many more were remarkable for a want of disturbance in the circulation proportionate to the other symptoms; some had an attendant cough, others, even where the side was excruciatingly painful, had no cough; some were attended with expectoration, most were without it, in a considerable degree. In fine, such was the discordance in the duration and symptoms of the cases of this complaint, that many could hardly be ranked under it, for any better reason, than that they differed from all other diseases. If we were called on to fix on any one external symptom symptom as most characteristic of the complaint, it would be the low state of the circulation, compared to its exaltation in most other diseases, with equally violent symptoms.

In order to prevent any misconception, it is proper to state, that the remarks made above were drawn from a careful observation of a few severe cases, and a great number of slight ones in the town of Boston.

The cause which *disposes* to this disease exists in the atmosphere. The *exciting causes* are, exposure to cold and moisture, and fatigue, especially after excesses and debauchery of any kind. So many extraordinary things have been thrown out as to the *proximate cause*, that we are unwilling to run the risk of incurring ridicule, by adding to the number. It seems necessary, however, to direct our attention to two or three remarkable circumstances, which may serve as a foundation for the conjectures of the ingenious. 1st. The part or texture primarily and mostly affected in this disease, is the *serous membrane*. When the thorax is attacked, the pleura, especially of the lungs, is diseased, or, in certain cases, the pericardium; when the abdomen, the peritoneum is deranged; and in the head, it is the tunica arachnoides. After the complaint has continued some time, the substance of the lungs gets disordered, and we see it swelled, hardened, and full of blood; and sometimes we have observed a redness of the mucous membrane of the bronchiæ. But these seem to be secondary derangements, for they are not observed in any organ where the serous membrane is not diseased, while the latter is often greatly disordered without any change in the organ it invests. In no instance has the substance of the brain been seen diseased, even when its membrane was most considerably affected, nor any of the abdominal viscera, when the peritoneum was diseased. The heart has been thought to be swelled in one or two instances, but this is an appearance difficult to judge of satisfactorily. The morbid appearances, besides those of the serous membrane, are therefore confined to the lungs. In that organ, the hardness and redness of its substance has been observed, in some instances, to be confined to small portions connected with inflamed spots in the pleura, and in others, where the membrane was uniformly affected, the swelling existed principally near the surface of the organ, from which we would infer, that the inflammation affected the pleura first, and extended from it to the lungs. 2d. The livid colour, the disposition to gangrene and sloughing, and the low state of the power of the heart, distinguish this inflammation of the serous membrane from common inflammation. It seems to be an inflammation peculiar to this disease.

These

These appearances serve to identify the disease with what was called petechial fever, in 1810. The apparent differences arise from the head having been most affected in the epidemic of 1810, and the lungs in that of the present season. In the former, however, the lungs were sometimes the principal seat of disease; as may be seen exemplified in case 11th, in the Report of the Massachusett's Medical Society: the morbid appearances of which precisely resembled those lately observed. While, in the course of the recent epidemic, various instances of morbid appearances in the head have been witnessed; and the symptoms in many of the most violent cases bear the closest resemblance to those of the disease of 1810. In both, the patients have been struck down suddenly as by light; in both, the head was first affected, when the disease was very violent; in both, the circulation was free from a disturbance proportionate to the other symptoms; the symptoms of different cases were equally variable from each other, and the progress in the same case equally irregular; the texture of the organ affected was the same, and often the organ itself was the same, and the appearance of inflammation peculiar, and alike in both. The manner in which the two disorders are affected by remedies, is certainly not similar. The epidemic of 1810 would not bear evacuations in any proportion of cases to that of 1813. Evacuations of some kind were always necessary and beneficial in the latter; but in the former, all evacuations were sometimes injurious, and always required to be very sparingly and cautiously employed. The dissimilarity in this respect, does not, however, show the two diseases to be of different character; as many, perhaps most epidemics, vary as much as these in the manner in which they are influenced by medicine. Cynanche maligna bears an inflammatory character in some seasons; but, in others, a putrid one: it sometimes requires evacuations, which at others are deleterious. In the symptom which gave name to the disease of 1810, the petechial eruption, this disease is entirely deficient. Some persons may be disposed to consider this as a very important difference, which ought to form a line of distinction between the two complaints; but those who saw much of the epidemic of 1810, are aware that the number of cases which exhibit genuine petechiæ is extremely small, and that this very interesting and formidable appearance was in most places so rare, as to be considered an object of curiosity. Its employment to designate the disease must, therefore, be considered very unfortunate.

The epidemic, of which we speak, is not contagious.

No specific medicine or mode of practice has been found appropriate to the cure of this disorder. Great contentions have existed as to the utility of bleeding on the one side, and the use of stimulants on the other; but the truth is, that the character of the disease is so infinitely various, that the treatment, to be successful, must be equally varied, and adapted, with cautious discrimination, to the existing state of symptoms. When the violence of local pain indicates the certainty of local disease, if the pulse is not too much depressed, bleeding may be considered the first and best remedy. It must be moderate, and cannot be repeated, in true cases of the epidemic, more than once or twice. In the most violent cases, bleeding, in the first stage, would prove fatal; and in many others, the pulse is too weak to justify it. It may be laid down as a general rule, that when the pulse will bear it, this remedy will be highly beneficial. Should the patient be depressed by it, moderate cordials may be employed temporarily. In no other circumstances has the use of cordials or stimulants been indicated during the present season, if we except some of those violent cases which proved fatal in a few hours. After bleeding, emetics of ipecacuanha with calomel will be found useful, provided the nausea of the stomach indicates them. When employed under that circumstance, there has usually been an evacuation of very thick green-coloured mucus; but when this remedy was not called for, a cathartic remedy has been preferred, and has usually produced discharges of the same green-coloured mucus of thinner consistence, and almost always with a degree of relief. No one, perhaps, will hesitate, in a disease commonly attended with such remarkable local affection, to employ blistering speedily, extensively, and repeatedly. Its effects are admirable. Small doses of calomel with opium and the tartrite of antimony, squills and seneka, are the best medicines for promoting expectoration. The gums have rarely become decidedly affected by calomel, so that the disorder cannot be set down as one of those readily cured by the preparations of quicksilver. As soon as the violence of the disease is subdued, tonics should be employed with due caution. The most suitable seems to be cascarilla employed in an infusion. The want of the critical expectoration which takes place in common pleurisy, is, perhaps, the cause of a dry and uncomfortable cough remaining after the bad symptoms have subsided, from the irritation produced by vessels not yet emptied of the blood they had received. In this state of things, opium is the best medicine; in other stages of the complaint, given in small doses, it has appeared to be at least a safe, and sometimes a useful, remedy.

The morbid appearances after death need not be generally stated, stated, as the two following cases will sufficiently exemplify them. The first of these cases affording a specimen of the most common and least remarkable appearances, and the second of the most extraordinary changes.*

The first was a soldier of a robust frame and ample chest, who, as well as his comrades, was in the habit of using ardent spirits very freely, and was lodged in a dirty and uncomfortable situation. He was suddenly attacked with pain in the right side, a severe stricture across the chest, and difficult respiration, with very slight cough. The pulse rather more frequent than natural, and full, but very compressible. Heat of the skin above the natural state. He was bled, with temporary relief from pain; but the bleeding was not repeated, nor did he have other medicine than calomel and opium. On the second day, his countenance was very livid, and his brain disturbed. On the third, the respiration was very laborious, pulse quicker and smaller; there was great restlessness, and complete delirium. On the fourth, he died.

Although I did not attend this patient, an opportunity was afforded me of examining the body very carefully. In the brain there was no very remarkable change of structure. The superficial veins were rather full; the little vessels of the medullary substance charged with blood; the plexus choroides was quite swelled, and contained a little water, and its interposed membrane was remarkably tough. When the thorax was opened, the heart and left lung were seen to be of a tolerably healthy appearance; but the right lung was very livid in every part, especially on the anterior surface. Its pleura was completely discoloured by blood in the small vessels, which rendered it black, in some parts, and the appearance of the pleura lining the ribs was similar. Over the surface of the lung was spread a thin layer of lymph, which, entering between the lobes, glued them together. The substance of the lung was hard, for some little distance from the surface, but the central part softer. This organ was generally of a dark colour within. The abdominal viscera exhibited no vestige of disease. The stomach contained a little watery fluid, and its mucous coat was reddened. The intestinal canal appeared quite healthy. The liver was very large, and rather harder than common. The gall-bladder large, full of bile of a good appearance, and the ducts quite pervious.

The second patient was a citizen, whom I did not visit till after he had been ill a few days, when he was evidently be-

* These dissections were made by Dr. J. C. Warren.

yond the reach of medical aid. His story was, that he was first attacked with chills, pain in the forehead and side; for which he employed liberal potations of hot brandy and water, or gin and water. Afterwards he found that his bowels became painful, and, when I saw him, it was difficult for him to determine in what part he suffered most severely; though he seemed disposed to consider the abdominal pains to be most distressing. The region of the abdomen was extremely tumid, and quite tender. The pulse was depressed, so that at times it could scarcely be perceived. The extremities were cold and damp. There was a stricture, like a cord, over the breast, a slight cough, and very difficult respiration. The tongue was black and dry. The eyes languid and sunk. In this state he continued eight days longer, his distress being occasionally relieved by opium. The functions of the brain were not greatly impaired till three days before death, when he became insensible.

The body was examined the day after death. The brain was very firm. Its superficial veins were shrunk, but exhibited satisfactory marks of having been much distended. The tunica arachnoides had many spots of coagulated lymph, by which the hemispheres were more closely connected than common. Some water was found in the ventricles. The heart was in a sound state. The left lung much discoloured, and partially hardened. The right lung was discovered lying in the upper part of its cavity, covered by yellow coagulated lymph, its substance remaining entire. The lower part of the same cavity was occupied by a kind of cyst formed by a recent concretion of lymph, about a quarter of an inch thick, on all sides, soft, and readily torn; and enclosing, in its cavity, more than three quarts of thick and discoloured serum. The anterior surface of the abdominal organs, the liver, spleen, stomach, and intestines, was covered with a most extraordinary thick coat of very yellow lymph, which cemented all these parts into one mass, and united them to the anterior parietes of the abdomen. This lymph was, in many places, an inch thick, and might be separated in large masses. The whole peritoneal surface of the organs, and of the parietes of the abdomen, was in a greater or less degree covered with lymph. By the union of the different organs, the appearance of these parts was rendered as confused as is possible to conceive. In various places, the adhering parts formed cysts of different sizes, which were usually filled with serum containing a portion of semi-purulent lymph. The peritoneum and pleura were generally very tender, and in some parts had the aspect of approaching gangrene.— *New-England Journal.*

